# Impact on the time elapsed since SARS-CoV-2 infection, vaccination history, and number of doses, on protection against reinfection

**DOI:** 10.1038/s41598-023-50335-6

**Published:** 2024-01-03

**Authors:** Laura Sánchez-de Prada, Ana María Martínez-García, Belén González-Fernández, Javier Gutiérrez-Ballesteros, Silvia Rojo-Rello, Sonsoles Garcinuño-Pérez, Alejandro Álvaro-Meca, Raúl Ortiz De Lejarazu, Iván Sanz-Muñoz, José M. Eiros

**Affiliations:** 1 Faculty of Medicine, University of Valladolid, Valladolid, Spain; 2 National Influenza Center of Valladolid, Valladolid, Spain; 3 Department of Microbiology and Immunology, Hospital Clínico Universitario de Valladolid, Valladolid, Spain; 4 Department of Preventive Medicine and Public Health, Rey Juan Carlos University, Madrid, Spain; 5 Centro de Investigación Biomédica en Red de Enfermedades Infecciosas (CIBERINFEC), Instituto de Salud Carlos III, Madrid, Spain

**Keywords:** Preventive medicine, Infectious diseases, Vaccines

## Abstract

SARS-CoV-2 reinfections have been frequent, even among those vaccinated. The aim of this study is to know if hybrid immunity (infection + vaccination) is affected by the moment of vaccination and number of doses received. We conducted a retrospective study in 746 patients with a history of COVID-19 reinfection and recovered the dates of infection and reinfection and vaccination status (date and number of doses). To assess differences in the time to reinfection(t_RI_) between unvaccinated, vaccinated before 6 months, and later; and comparing one, two or three doses (incomplete, complete and booster regime) we performed the log-rank test of the cumulative incidence calculated as 1 minus the Kaplan–Meier estimator. Also, an adjusted Cox-regression was performed to evaluate the risk of reinfection in all groups. The t_RI_ was significantly higher in those vaccinated vs. non-vaccinated (p < 0.001). However, an early incomplete regime protects similar time than not receiving a vaccine. Vaccination before 6 months after infection showed a lower t_RI_ compared to those vaccinated later with the same regime (adj-p < 0.001). Actually, early vaccination with complete and booster regimes provided lower length of protection compared to vaccinating later with incomplete and complete regime, respectively. Vaccination with complete and booster regimes significantly increases the t_RI_ (adj-p < 0.001). Vaccination increases the time it takes for a person to become reinfected with SARS-CoV-2. Increasing the time from infection to vaccination increases the time in which a person could be reinfected and reduces the risk of reinfection, especially in complete and booster regimes. Those results emphasize the role of vaccines and boosters during the pandemic and can guide strategies on future vaccination policy.

## Introduction

Until mid-October 2023, the COVID-19 pandemic has been responsible for more than 770 million cases and nearly 7 million deaths^1^. Different vaccine approaches against COVID-19 arrived and evolved along with the virus through the pandemic^2–4^. The fast vaccine development has made possible for developed countries to reach a considerable vaccine coverage in an amazing short period of time^5^. However, concerns related to side effects and changes in commercialization authorizations, have caused delays in the administration of second doses, heterologous vaccination, and infections at the time of vaccination. In addition, the continuous raise of variants of concern (VOC) and their spread across the world^6^, have led to a variable viral immunoescape to antibodies elicited by vaccines. Thus, viral evolution has led to breakthrough with VOCs in vaccinated populations^7–9^. Those factors have caused for some individuals to present a different immune status that has been called hybrid immunity.

It has been postulated that a more robust immune response is obtained by vaccination before or after SARS-CoV2 infection^10^. As VOC keep emerging and vaccines evolving, more concerns about immune escape after infection or vaccination with the original strain arise^11^. Actually, vaccine breakthrough has been documented since early stages of the pandemic and have surged, especially after the emergence of some variants^12,13^, becoming more often detected by the National Influenza Centers (NICs) as a part of the Global Influenza Surveillance and Response System (GISRS) which integrates COVID-19 and Influenza surveillance among other roles^14^.

Different circumstances influencing breakthrough after a first infection and subsequent vaccination, so called hybrid immunity breakthrough infection (HIBI), have been explored, particularly those involving time of vaccination. The results obtained in this study show different protection patterns of hybrid immunization associated to different vaccine schedules, boosters, and time of vaccination, and help guide future strategies on COVID-19 vaccination.

## Methods

### Study design and materials

**Figure 1:**
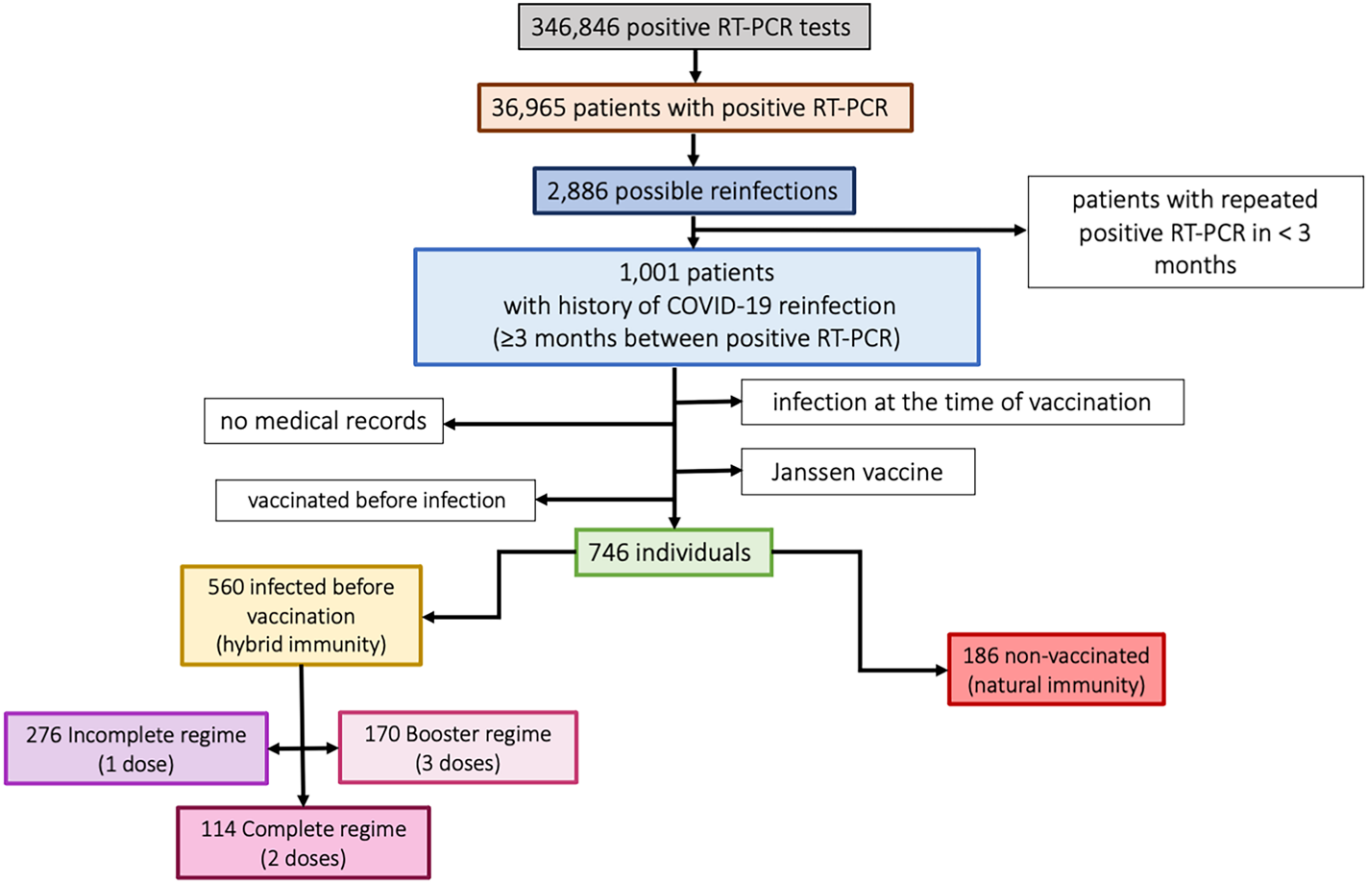
Diagram of selection criteria for individuals in the study.

A retrospective observational study was performed by the National Influenza Centre (NIC) in collaboration with the Microbiology department at the Hospital Clínico Universitario of Valladolid, Spain. Data was extracted from the laboratory database of 346,846 positive RT-PCR tests to confirm infections between March 2020 and April 30th, 2022. Due to scarce availability of tests at the beginning of pandemic and other circumstances, different PCR tests were employed, namely Roche (Switzerland), Vircell (Spain), Vitro (Spain), Cepheid (USA)m, Menarini (Italy), and Thermofisher (USA). All of them detected at least two genes and were considered positive following manufacturer criteria. By looking for reinfection, a total of 2886 patients with history of COVID-19 reinfection were initially selected from 36,965 patients with positive tests. As initial exclusion criteria, patients whose laboratory confirmed infections were less than three months apart were discarded and considered to be the same infection episode^15^ (Fig. 1). Dates of first and second infection were collected, as well as vaccination status and if such, date of vaccination of correspondent doses and vaccine used. Then, other exclusion criteria were applied: patients with no vaccine details in their medical records, patients who had the infection at the time of vaccination or patients with no prior history of COVID-19 infection before immunization. At the beginning of 2021, 4 different vaccines were authorized in all EU countries. Three vaccines in which two doses were required for full immunization: Comirnaty (Pfizer®), Spikevax (Moderna®) and Vaxzevria (AstraZeneca®) with intervals between doses of 3 weeks, 4 weeks, and 8–12 weeks, respectively. And one vaccine in which only one dose was required: COVID-19 vaccine Janssen (Janssen®), therefore individuals receiving that vaccine were discarded.

A total of 746 cases with history of repeated COVID-19 infection were included and 560 of them had HIBI. The 186 individuals who did not receive any vaccine but suffered reinfection were considered as controls. From patients that presented HIBI, individuals were classified according to their vaccine status: 276 had received one dose of vaccine (incomplete vaccine regime), 114 two doses (complete vaccine regime), and 170 three doses (booster vaccine regime). Different vaccine regimes schedules were due to changes in vaccine guidelines as the pandemic evolved^16^ (Fig. 1).

.

### Parameters and definitions

**Figure 2:**
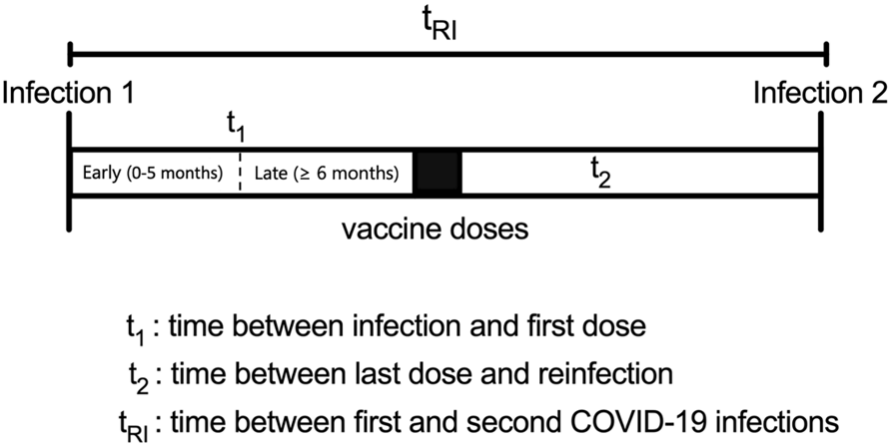
Diagram of the different time parameters analyzed in the study of reinfection.

To evaluate HIBI, only patients who were first infected and then vaccinated were taken into consideration. Three different parameters to assess reinfection and HIBI were considered. First, the time elapsed between first COVID-19 infection and first dose of vaccination (t_1_) which was used to divide the different regime cohorts in two: early when it was received in the first five months post-infection, and late when it was received after/equal six months (Fig. 2). Then, t_2_ represents the time elapsed between the last dose of vaccine received and the second COVID-19 infection (HIBI). And finally, t_RI_ or time of reinfection represents the time elapsed between first infection and reinfection, independently of receiving a vaccine in the meantime. Time was measured in months to help perform the analysis.

A timeline of the daily diagnosed cases and the variants that circulated in the Spanish region studied was created in Supplementary Fig. 1 to represent the evolution of the pandemic during the study period.

### Statistical analysis

Initially, t_2_ and t_RI._ were calculated as median (interquartile range, IQR). A Cox-regression adjusted by sex, age, heart disease, lung disease, hypertension, immunosuppression, and diabetes mellitus, was performed in order to evaluate the risk of reinfection in vaccinated groups. Differences in t_2_ and t_RI_ between vaccinated and non-vaccinated were assessed using the two-tailed Mann–Whitney U-test. The Cumulative Incidence of Covid-19 reinfection of different cohorts was calculated as 1 minus the Kaplan–Meier estimator. Difference between cumulative incidence was assessed by log-rank test. Additionally, individual comparisons between groups were calculated with the same test correcting for multiple comparisons with the False Discovery rate. GraphPad Prism Version 9 (GraphPad Software, San Diego, CA, USA) and R software, version 4.2.1(GNU- General Public License, the R Core Team, R, 2022). p values < 0.05 were considered statistically significant.

### Ethical statement

This research was performed according to the Declaration of Helsinki. The database was anonymized and the clinical data for this observational study was performed under the strict fulfillment of the Spanish Organic Law 41/2002 for regulation of patient’s autonomy and his rights and obligations in matters of information and clinical documentation (BOE nº 274, 15th November 2002). This research was approved by the Ethics Committee of East-Valladolid health area under the code PI 22-2920. The informed consent was waived by the Ethics Committee of East-Valladolid health area due to the retrospective nature of the study and the anonymous nature of the dataset.

## Results

### Profile of patients who suffered COVID-19 reinfection

Patients who suffered reinfections (746) were divided in four groups according to vaccine status: non-vaccinated (used as controls) (n = 186, 24.9%), reinfected after incomplete vaccine regime (n = 276, 37.0%), reinfected after a complete vaccine regime (n = 114, 15.3%) and reinfected after a booster regime (n = 170, 22.8%). In addition, vaccinated individuals with all regimes were then divided depending on when the vaccine was received: in the first five months post-infection (early dose) or after/equal six months (late dose). The characteristics are described in Table 1.

**Table 1:** Patient characteristics and time of reinfection (t_RI_) and time from the last dose (t_2_).

			Incomplete regime		Complete regime		Booster regime	
	Total	Non vaccinated	Early 1 dose	Late 1 dose	Early 2 doses	Late 2 doses	Early 3 doses	Late 3 doses
No	746	186	65	211	57	57	105	65
Sex (male) (n, %)	305 (40.9)	88 (47.3)	28 (43.1)	90 (42.7)	16 (28.1)	26 (45.6)	31 (29.5)	26 (40.0)
Age (years) (median, IQR)	42.0 (27.0; 62.0)	29.5 (15.0; 50.0)	27.0 (21.0; 36.0)	37.0 (27.0; 47.0)	53.0 (39.0; 77.0)	50.0 (32.0; 67.0)	82.0 (56.0; 89.0)	69.0 (46.0; 87.0)
Time since first infection (months) (median, IQR)	14.0 (9.0; 16.0)	6.0 (4.0; 10.0)	6.0 (5.0; 9.0)	14.0 (13.0; 15.0)	11.0 (9.0; 14.0)	16.0 (15.0; 17.0)	15.0 (14.0; 17.0)	21.0 (20.0; 22.0)
Time after last dose (months) (median, IQR)	4.0 (3.0; 6.0)	–	3.0 (3.0; 5.0)	5.0 (4.0; 6.0)	7.0 (4.0; 9.0)	5.0 (3.0; 8.0)	4.0 (2.0; 5.0)	4.0 (1.0; 4.0)
Clinical characteristics (n, %)								
Hypertension	151 (20.2)	25 (13.4)	0 (0.0)	20 (9.5)	15 (26.3)	8 (14.0)	61 (58.1)	22 (33.8)
Heart Disease	70 (9.4)	10 (5.4)	1 (1.5)	6 (2.8)	6 (10.5)	4 (7.0)	23 (21.9)	20 (30.8)
Immunosupression	74 (9.9)	20 (10.8)	3 (4.6)	16 (7.6)	6 (10.5)	6 (10.5)	13 (12.4)	10 (15.4)
Lung disease	81 (10.9)	21 (11.3)	4 (6.2)	20 (9.5)	5 (8.8)	5 (8.8)	13 (12.4)	13 (20.0)
Diabetes mellitus	56 (7.5)	8 (4.3)	0 (0.0)	8 (3.8)	7 (12.3)	5 (8.8)	17 (16.2)	11 (16.9)

No, number; IQR, interquartile range.

Globally 84.15% of the doses were Comirnaty (Pfizer®), 12.35% were Spikevax (Moderna®), and 3.5% were Vaxzevria (AstraZeneca®). Only, 69 patients received a heterologous schedule combining more than one vaccine in any of the subsequent doses.

Regarding comorbidities, the Cox regression indicated that age could influence the risk of reinfection. Also, patients with immunosuppression and diabetes mellitus had an increased risk of suffering a reinfection with an adjusted Hazard Ratio (aHR) of 1.45 (p = 0.005) and 1.39 (p = 0.028), respectively. (Supplementary Table 1).

### Vaccination provides longer-lasting protection against reinfection

**Figure 3:**
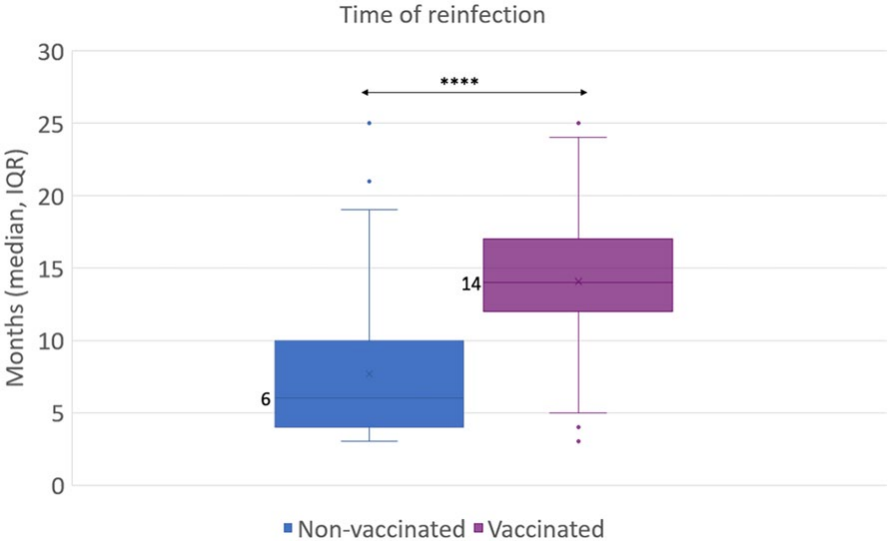
Time of reinfection (t_RI_). The time elapsed between first and second COVID-19 infection and the comparison between vaccinated (with either incomplete, complete or booster regime) and non-vaccinated is represented. The two-tailed p-value was calculated by applying Mann–Whitney U-test; ***p < 0.001.

We next analyzed the different times of reinfection between vaccinated population and non-vaccinated population. The median time from infection to reinfection (t_RI_) in non-vaccinated population was 6 months (IQR: 4–10), being significantly lower compared to 14 months (IQR: 9–16) in individuals that received either one, two or three doses of vaccine after their first infection (p < 0.001) (Fig. 3). However, the risk of reinfection was higher in those receiving one early vaccine dose (Table 2).

**Table 2:** Adjusted Hazard ratio (aHR) calculated for the time of reinfection (t_RI_) of different vaccine groups.

	Groups	T_RI_	
		aHR (CI95%)	p-value
Incomplete regime	Early 1 dose	2.11 (1.56–2.84)	< 0.001
	Late 1 dose	0.35 (0.28–0.43)	< 0.001
Complete regime	Early 2 doses	0.73 (0.53–1.01)	0.057
	Late 2 doses	0.17 (0.12–0.24)	< 0.001
Booster regime	Early 3 doses	0.20 (0.15–0.27)	< 0.001
	Late 3 doses	0.05 (0.04–0.07)	< 0.001

p-values were calculated by a Cox regression.

### Vaccination from 6 months after infection could provide 6 more months of protection

**Figure 4:**
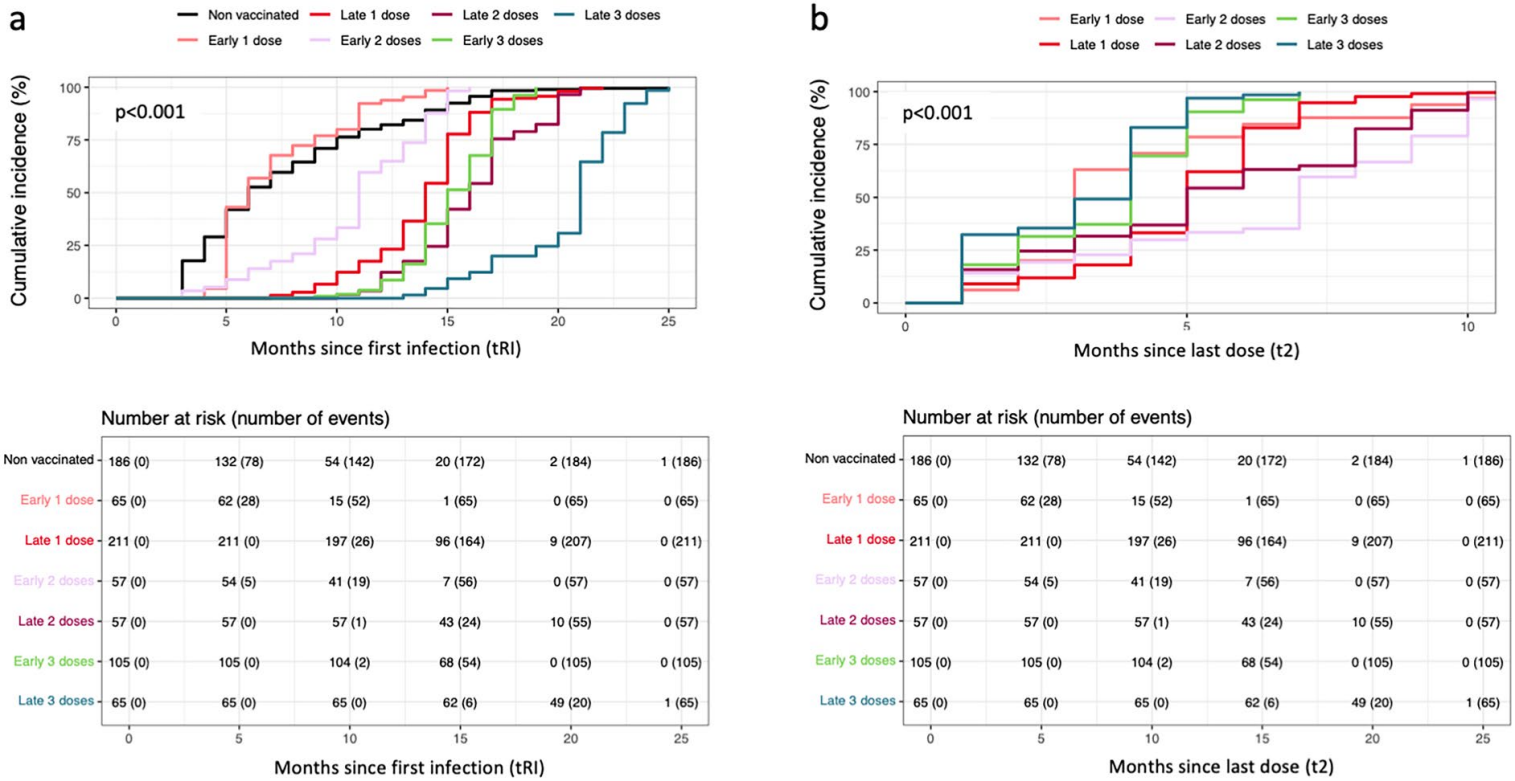
(**a**) The Cumulative Incidence of time between both Covid-19 infections (t_RI_) of different cohorts was represented as 1 minus the Kaplan–Meier estimator. Difference between cumulative incidence was assessed by log-rank test. (**b**) The Cumulative Incidence of time between last dose and reinfection (t_2_) of different cohorts was represented as 1 minus the Kaplan–Meier estimator. Difference between cumulative incidence was assessed by log-rank test.

Here the time of HIBI after the last vaccine dose (t_2_) and the total time between both first infection and the reinfection (t_RI_) were analyzed. Based on t_1_ being under or over/equal to six months, we considered those who received an early or a late first dose. Significantly higher t_2_ was found in those who received a late first those with a median of 5 months (IQR:3–6) compared to 4 months (IQR: 2–6) in those vaccinated earlier (FDR adjusted p = 0.034). Again, higher t_RI_ was found in those who waited with a median of 15 months (IQR: 13–17) compared to 12 months (IQR: 8–15) in those vaccinated earlier (FDR adjusted < 0.001). Then, based again on that t_1,_ incomplete, complete and booster regime cohorts were divided into early one dose, late one dose, early two doses, late two doses, early three doses, and late three doses. Then, to explore the differences between incomplete, complete and booster regimes and their schedules, cumulative incidences were calculated, and differences between cohorts were computed as well (Fig. 4 and Table 2).

First, the highest t_RI_ was found in individuals who received a booster regime after the initial two doses, the first one of them received at or after six months from COVID-19 infection (FDR adjusted p < 0.001). The lowest value was found in individuals that received an early incomplete regime which was similar to that of non-vaccinated individuals, with a median t_RI_ of 6 months (FDR adjusted p = 0.849). In fact, the Cox regression showed a doubled risk of reinfection in this group compared to non-vaccinated group (aHR = 2.11, p < 0.001) (Table 2). The rest of the cohorts showed significantly higher t_RI_ compared to non-vaccinated individuals (FDR adjusted p < 0.001) (Fig. 4a) as well as lower risk of reinfection (Table 2).

Then, the comparison regarding t_1_ in each regime (incomplete, complete and booster) showed that receiving the first dose at a minimum of 6 months post-infection provided a significantly higher t_RI_ (median time 14, 16 and 21 months) compared to receiving it earlier (median time 6, 11 and 15 months) (FDR adjusted p < 0.001 for all three comparisons) (Fig. 4A). Interestingly, the time from last dose of vaccine until the HIBI event (t_2_) was not significantly different when comparing schedules of the same regime (Fig. 4b).

### Better late than ever, even with an incomplete vaccine regime

Next, we decided to compare the differences in the time parameters analyzed between the people vaccinated with different regimes. In those who have received the vaccine earlier after infection, higher t_RI_ was found in the booster regime with a median time of 15 months (IQR: 14–17) compared to the other two (FDR adjusted p < 0.001). Additionally, t_RI_ in the early complete regime group (11 months, IQR: 9–14) was higher than the early incomplete one (6 months, IQR: 5–9) (FDR adjusted p < 0.001). Similar results were found in those who receive a late dose post-infection. The highest t_RI_ was found in the booster regime (21 months, IQR 20–22) which was significantly higher than complete (16 months, IQR 15–17) and incomplete (14 months, IQR 13–15) regimes (FDR adjusted p < 0.001). Also, complete regime showed higher t_RI_ than incomplete regime (FDR adjusted p < 0.001). Remarkably, t_RI_ in individuals receiving a late 1 dose (late incomplete) and late 2 doses (late complete) were significantly higher when compared to early 2 doses (early complete) and early 3 doses (early booster), respectively (Fig. 4). The Cox-regression showed higher risks of reinfection in early compared to late vaccination with the same number of doses, remasrking the importance of taking an appropriate time for vaccination after infection. Additionally, the lowest risks of reinfection with a reduction of at least 80% compared to non-vaccinated individuals was found in those with a complete late regime or any booster regime (Table 2).

Regarding t_2_, a higher value was found in early 2 doses group when compared to early doses of the incomplete regime and the booster regime groups (FDR adjusted p < 0.001). Additionally, when comparing late doses, t_2_ was lower in the booster regime compared to both, the complete and incomplete regime (FDR adjusted p < 0.001). Although median times were similar, in the case of the late incomplete regime, the cumulative incidence is much higher than in the complete one, from the fifth month on (FDR adjusted p = 0.014).

## Discussion

As far as we know, this is the first study to analyze deeply HIBI in Spain, and although there are several COVID-19 reinfection publications^17–19^, none of them have yet described HIBI based on immunization regimes and schedules. This study integrates laboratory testing and immunization registry and reinfection data since the beginning of the COVID-19 pandemic along different variants emerged in Spain and so in Europe.

Three important points arise from the results of this study. First, vaccination after infection offers more protection than not getting vaccinated after infection, except for an early incomplete regime, in patients who got reinfected. Our results show that natural infection prevents significatively reinfection during a median period of 6 months. Secondly, complete and booster vaccine regimes in individuals that have previously been infected indeed confers a significant benefit by prolonging the time of reinfection compared to individuals with an incomplete regime. Third, data showed that vaccination too close after infection (minor than six months), despite the regime used, decreases significatively the time of reinfection.

Pre-existing immunity against SARS-CoV-2 through vaccination or infection is characterized by robust immune responses that had previously been associated with protection against infection or severe disease. However, as countries have reached significant vaccine coverage among their populations, infections in vaccinated individuals have been observed, leading to the concept of hybrid immunity^20–22^. A kind of immune response characterized by vaccination plus natural infection of virus variants. This is a very important aspect when a new virus of wide diffusion emerges into a naïve population and imprints their immune system, driving the future evolution of the virus in populations according to their vaccine coverage rates. The results showed that reinfection takes place earlier in non-vaccinated individuals compared to vaccinated, and vaccination itself increases protection against reinfection up to 14 months compare to a median of 6-month protection provided by the first infection. Our results are aligned with previous studies indicating that hybrid immunity seems to protect against reinfection longer than just natural acquired immunity^10,23,24^. In respiratory transmitted infections by variable viruses, this global effect of hybrid immunity has not been previously considered and could change future vaccination schedules based on natural exposure.

Initially, studies showed that population previously infected by SARS-CoV-2 tend to mount intense immune responses with a single shot reaching levels equal or greater than naïve individuals with two doses^25–27^. Based on that, vaccine recommendations on these patients were to have only one dose in the urge to have more doses to vaccinate and reach herd immunity^16^. Our results show that having an incomplete vaccine regime does not provide longer protection against reinfection. Interestingly, despite the current debate about boosters, in previously infected individuals, claiming they could be unnecessary^28^, our results prove the protection benefit acquired after a complete and a third-booster regime even after natural infection. Returning to the herd immunity concept we must consider that, similarly to other non-viremic respiratory infections that do confer low or no long protection against reinfection^29^, a different epidemiological concept view of herd immunity is applied. This is reaching the level of immunity in a population where no additional prophylactic measures add significant protection. However, with the spread of omicron subvariants the debate still goes on^30^. In Spain as well as in other European countries a third dose or booster regime was initially recommended for patients at higher risk, starting with institutionalized patients in September 2021, followed by the elderly and sanitary workers through autumn that year. There has an ongoing debate whether age is a factor affecting vaccine efficacy, but it has been shown that actually frailty in aged population is what leads to weaker immune responses^31^ which relates with age being a factor in the risk of reinfection.

When Omicron variant was introduced in Spain in December 2021, its circulation was predominant by the end of the month and later through January 2022, with an exponential increase of cases^32^. Our results show individuals infected after three doses, despite having a longer time to reinfection, have lower times from the last protecting event. Many factors affected this. First, many individuals did not have the right amount of time to mount appropriate responses. Second, omicron variants are known to evade the immune system to a certain extent. Although booster regimes have shown to increase protection, it wanes faster compared to previous variants^33^. In fact, that time has been estimated to be three months, similar to our results^33^. Finally, booster doses were based on the initial wild-type SARS-CoV-2 variant that emerged in Wuhan, which would mount antibodies with a reduced neutralizing capacity to omicron variants^32^. Actually, it has been suggested that an *original antigenic sin* effect, similar to that found in influenza virus^34^, might shape humoral immune responses to new variants by eliciting greater responses to those first variants encountered in life^35^. Hence, vaccine updates might be periodically needed to improve responses to mutated variants rising.

Other factor influencing HIBI is the time interval between infection and vaccination, regardless of the number of doses received. Initial vaccination guidelines moved from disregarding time to vaccine after infection, to recommending waiting a minimum of five to six months^16^. Previous studies of vaccines against different pathogens revealed spacing between doses positively influence vaccine responses. Actually, schedules with longer intervals tend to lead to increased immune responses than accelerated schedules^36^. In this way, our work confirms that vaccination too close to the infection negatively affect the immune response by shortening the time of reinfection. Thus, the protection time provided by vaccination after a minimum of six months infection was significantly higher than vaccination in the first five months after infection. This occurred independently of receiving an incomplete, complete or booster vaccination regime. Furthermore, late doses in incomplete and complete regimes provided longer protection compared to early doses in complete and booster regimes, respectively. Negative interference and reaching a “ceiling effect” could explain the above observation^37^. If a vaccine antigen antigenically close is introduced in an experienced individual, the higher level of pre-existing immunity against it, would rapidly clear the antigen instead of mounting new specific immune responses^37^. On the other hand, heterologous vaccination in a minimum number of individuals does not allow this work to detect differences between them.

Our study has some limitations. This is an observational retrospective study based on RT-PCR testing to confirm infection and reinfection, but no a cohorts’ study. We do not study risk factors or reinfection itself and we only address HIBI and time observed in those who get reinfected. We do not have information about the variants causing the infection or reinfection or the severity of the infection. For that reason, it is unknown to what extent viral variability and immune escape could acts as a factor related to time of reinfection.

In summary, vaccination against COVID-19 after infection increases the time of protection against reinfection in those who eventually will have a reinfection, highlighting the importance of vaccination, even in individuals previously infected. In those cases, time of reinfection relies upon two factors. First, number of doses received after infection, and. second, spacing out doses disregarding number of them, increases the time of reinfection.

The importance of this study lies in two main circumstances. First, this virus is here to stay circulating, probably for a while. Second, fully protective immune responses against infection tend to wane over time. Different and complex patterns of immunity are taking place in individuals and population; therefore, the results of this work can help design future vaccine strategies.

### Supplementary Information

Supplementary Figure 1.

## Data Availability

The dataset can be made available upon reasonable request to the corresponding author.

## References

[1] World Health Organisation (WHO). WHO Coronavirus (COVID-19) Dashboard [Internet] (2023, accessed 10 May 2023). https://covid19.who.int/.

[2] Voysey M, Costa Clemens SA, Madhi SA, Weckx LY, Folegatti PM, Aley PK (2021). Single-dose administration and the influence of the timing of the booster dose on immunogenicity and efficacy of ChAdOx1 nCoV-19 (AZD1222) vaccine: A pooled analysis of four randomised trials. The Lancet.

[3] Baden LR, El Sahly HM, Essink B, Kotloff K, Frey S, Novak R (2021). Efficacy and safety of the mRNA-1273 SARS-CoV-2 vaccine. New Engl. J. Med..

[4] Polack FP, Thomas SJ, Kitchin N, Absalon J, Gurtman A, Lockhart S (2020). Safety and efficacy of the BNT162b2 mRNA Covid-19 vaccine. New Engl. J. Med..

[5] Kirby T (2021). Has Spain reached herd immunity?. Lancet Respir Med..

[6] Salehi-Vaziri M, Fazlalipour M, Seyed Khorrami SM, Azadmanesh K, Pouriayevali MH, Jalali T (2022). The ins and outs of SARS-CoV-2 variants of concern (VOCs). Arch. Virol..

[7] Maggi F, Novazzi F, Genoni A, Baj A, Spezia PG, Focosi D (2021). Imported SARS-CoV-2 variant P.1 in traveler returning from Brazil to Italy. Emerg. Infect. Dis..

[8] Davis C, Logan N, Tyson G, Orton R, Harvey WT, Perkins JS (2021). Reduced neutralisation of the Delta (B.1.617.2) SARS-CoV-2 variant of concern following vaccination. Lee B, editor. PLoS Pathog..

[9] Kannan S, Shaik SAP, Sheeza A (2021). Omicron (B.1.1.529)—variant of concern—molecular profile and epidemiology: A mini review. Eur. Rev. Med. Pharmacol. Sci..

[10] Bates TA, McBride SK, Leier HC, Guzman G, Lyski ZL, Schoen D (2022). Vaccination before or after SARS-CoV-2 infection leads to robust humoral response and antibodies that effectively neutralize variants. Sci. Immunol..

[11] Chen X, Chen Z, Azman AS, Sun R, Lu W, Zheng N (2021). Neutralizing antibodies against SARS-CoV-2 variants induced by natural infection or vaccination: A systematic review and individual data meta-analysis. SSRN Electron. J..

[12] Nainu F, Abidin RS, Bahar MA, Frediansyah A, Emran TB, Rabaan AA (2020). SARS-CoV-2 reinfection and implications for vaccine development. Hum. Vacc. Immunother..

[13] Haque A, Pant AB (2022). Mitigating Covid-19 in the face of emerging virus variants, breakthrough infections and vaccine hesitancy. J. Autoimmun..

[14] World Health Organisation (WHO). Fact sheet: Global Influenza Surveillance and Response System (GISRS) [Internet] (2022, accessed 24 Oct 2023). https://www.who.int/initiatives/global-influenza-surveillance-and-response-system.

[15] National Center for Immunization and Respiratory Diseases(NCIRD), Division of Viral Diseases. Investigative Criteria for Suspected Cases of SARS-CoV-2 Reinfection [Internet] (2022, accessed 31 Jan 2022). https://www.cdc.gov/coronavirus/2019-ncov/php/invest-criteria.html.

[16] Grupo de Trabajo Técnico de Vacunación COVID-19 de la P de P y R de VacunacionesM de Sanidad. Principales cambios en las Actualizaciones de la Estrategia de Vacunación frente a COVID-19 en España. Actualizaciones 1–10. [Internet] (2022, accessed 31 Jan 2022). https://www.sanidad.gob.es/profesionales/saludPublica/prevPromocion/vacunaciones/covid19/Actualizaciones_EstrategiaVacunacionCOVID-19.htm.

[17] Lo Muzio L, Ambosino M, Lo Muzio E, Quadri MFA (2021). SARS-CoV-2 reinfection is a new challenge for the effectiveness of global vaccination campaign: A systematic review of cases reported in literature. Int. J. Environ. Res. Public Health.

[18] Wang J, Kaperak C, Sato T, Sakuraba A (2021). Covid-19 reinfection: A rapid systematic review of case reports and case series. J. Investig. Med..

[19] Vitale J, Mumoli N, Clerici P, De Paschale M, Evangelista I, Cei M (2021). Assessment of SARS-CoV-2 reinfection 1 year after primary infection in a population in Lombardy, Italy. JAMA Intern. Med..

[20] Wang Z, Muecksch F, Schaefer-Babajew D, Finkin S, Viant C, Gaebler C (2021). Naturally enhanced neutralizing breadth against SARS-CoV-2 one year after infection. Nature.

[21] Bates TA, McBride SK, Winders B, Schoen D, Trautmann L, Curlin ME (2022). Antibody response and variant cross-neutralization after SARS-CoV-2 breakthrough infection. JAMA.

[22] Rodda LB, Morawski PA, Pruner KB, Fahning ML, Howard CA, Franko N (2022). Imprinted SARS-CoV-2-specific memory lymphocytes define hybrid immunity. Cell.

[23] Kojima N, Shrestha NK, Klausner JD (2021). A Systematic review of the protective effect of prior SARS-CoV-2 infection on repeat infection. Eval. Health Prof..

[24] Pilz S, Theiler-Schwetz V, Trummer C, Krause R, Ioannidis JPA (2022). SARS-CoV-2 reinfections: Overview of efficacy and duration of natural and hybrid immunity. Environ. Res..

[25] Krammer F, Srivastava K, Alshammary H, Amoako AA, Awawda MH, Beach KF (2021). Antibody responses in seropositive persons after a single dose of SARS-CoV-2 mRNA vaccine. New Engl. J. Med..

[26] Ebinger JE, Fert-Bober J, Printsev I, Wu M, Sun N, Prostko JC (2021). Antibody responses to the BNT162b2 mRNA vaccine in individuals previously infected with SARS-CoV-2. Nat. Med..

[27] Goel RR, Apostolidis SA, Painter MM, Mathew D, Pattekar A, Kuthuru O (2021). Distinct antibody and memory B cell responses in SARS-CoV-2 naïve and recovered individuals after mRNA vaccination. Sci. Immunol..

[28] Clare Roth. COVID: Do multiple boosters “exhaust” our immune response? [Internet]. Deutsche Welle-Science. (2022, accessed 8 Feb 2022). https://p.dw.com/p/45dDT.

[29] Morens DM, Taubenberger JK, Fauci AS (2023). Rethinking next-generation vaccines for coronaviruses, influenzaviruses, and other respiratory viruses. Cell Host Microbe..

[30] He X, Su J, Ma Y, Zhang W, Tang S (2022). A comprehensive analysis of the efficacy and effectiveness of COVID-19 vaccines. Front. Immunol..

[31] Andrew MK, Shinde V, Ye L, Hatchette T, Haguinet F, Dos Santos G (2017). The importance of frailty in the assessment of influenza vaccine effectiveness against influenza-related hospitalization in elderly people. J. Infect. Dis..

[32] Centro de Coordinación de Alertas y Emergencias Sanitarias. Evaluación Rápida de Riesgo de Variantes de SARS-CoV-2 en España: linajes BA.2.12.1, BA.4 y BA.5 de Ómicron. 11^a^ Actualización. [Internet]. 2022. https://www.sanidad.gob.es/profesionales/saludPublica/ccayes/alertasActual/nCov/documentos/20220628-ERR.pdf.

[33] Andrews N, Stowe J, Kirsebom F, Toffa S, Rickeard T, Gallagher E (2022). Covid-19 vaccine effectiveness against the omicron (B.1.1.529) Variant. New Engl. J. Med..

[34] Francis T (1960). On the doctrine of original antigenic sin. Proc. Am. Philos. Soc..

[35] Ju B, Fan Q, Wang M, Liao X, Guo H, Wang H (2022). Antigenic sin of wild-type SARS-CoV-2 vaccine shapes poor cross-neutralization of BA.4/5/2.75 subvariants in BA.2 breakthrough infections. Nat. Commun..

[36] Zimmermann P, Curtis N (2019). Factors that influence the immune response to vaccination. Clin. Microbiol. Rev..

[37] Smith DJ, Forrest S, Ackley DH, Perelson AS (1999). Variable efficacy of repeated annual influenza vaccination. Proc. Natl. Acad. Sci. U. S. A..

