# Supplementary material for: Impact on the time elapsed since SARS-CoV-2 infection, vaccination history, and number of doses, on protection against reinfection
Source: Sci Rep. 2024 Jan 3;14:353. doi: 10.1038/s41598-023-50335-6 (PMC10764833; doi:10.1038/s41598-023-50335-6)
Supplement: Supplementary file 1 — Supplementary Figure 1. [file 41598_2023_50335_MOESM1_ESM.docx]

**SUPPLEMENTARY MATERIAL**

**Supplementary figure 1**. Timeline of daily cases and SARS-CoV-2 pandemic evolution in the region covered by the Hospital Clínico Universitario de Valladolid during the study period.


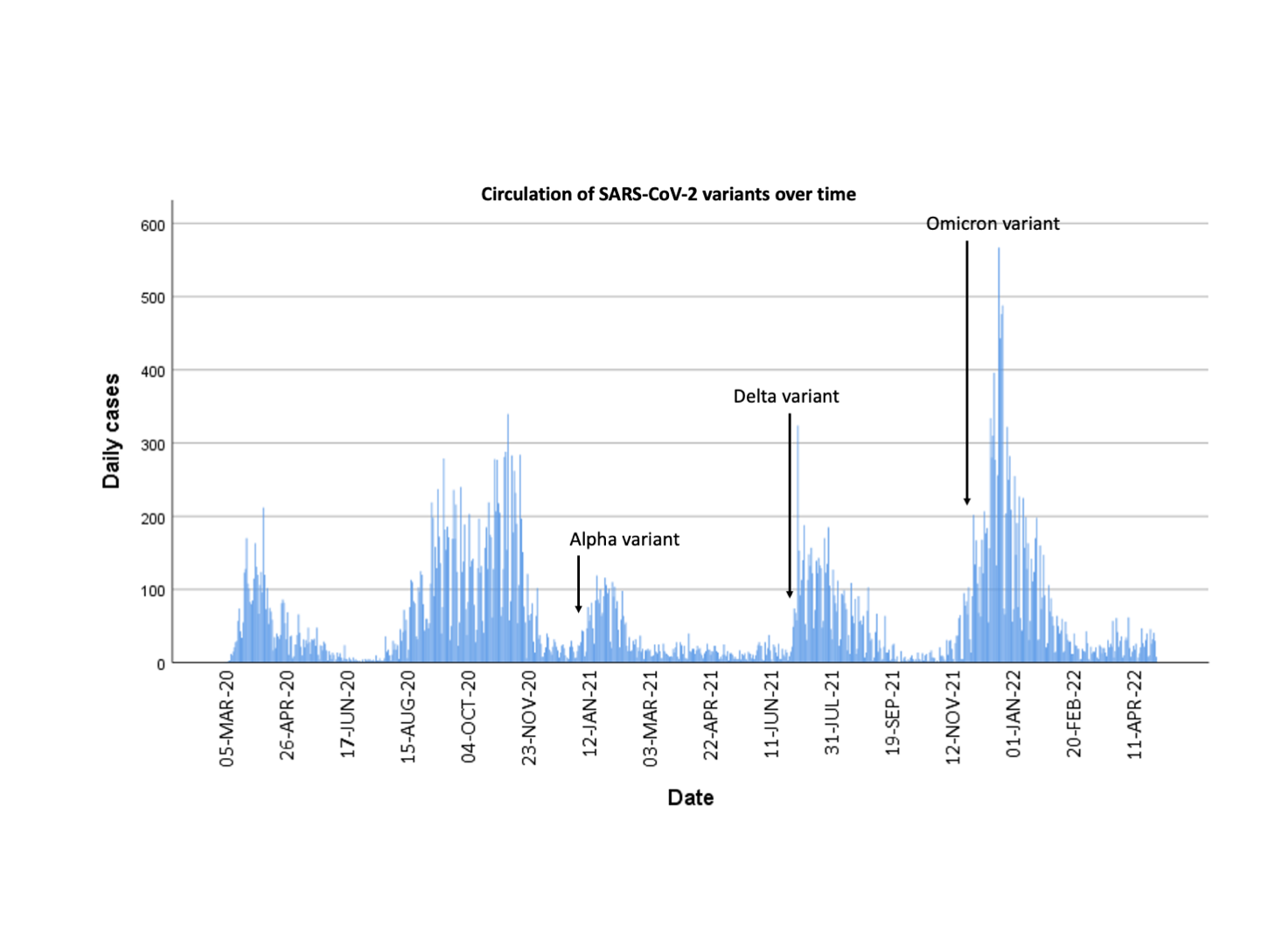


**Supplementary table 1.** Adjusted Hazard Ratio (aHR) calculated for the time of reinfection (TRI) adjusted for patient characteristics.

|  |  | **T_RI_** | |
| --- | --- | --- | --- |
|  | **Groups** | **aHR (CI95%)** | **p-value** |
| **Patient characteristics** | Sex | 0.97 (0.83-1.13) | 0.674 |
|  | Age | 1.00 (1.00-1.01) | 0.010 |
|  | Diabetes mellitus | 1.39 (1.04-1.88) | 0.028 |
|  | Hypertension | 0.89 (0.71-1.13) | 0.350 |
|  | Immunosupression | 1.45 (1.12-1.91) | 0.005 |
|  | Heart disease | 1.18 (0.9-1.56) | 0.237 |
|  | Lung disease | 0.89 (0.69-1.15) | 0.378 |

p-values were calculated by a Cox-regression.
